# Needs Assessment of Non-core Content for Podcasts in Emergency Medicine

**DOI:** 10.7759/cureus.47613

**Published:** 2023-10-24

**Authors:** Alvin Nagi, Andrew Little

**Affiliations:** 1 Emergency Medicine, Kansas Health Science Center - Kansas College of Osteopathic Medicine, Wichita, USA; 2 Emergency Medicine, AdventHealth East Orlando, Orlando, USA

**Keywords:** medical education, em podcasts, em, emergency medicine podcasts, emergency medicine

## Abstract

Study objective

The purpose of this study was to assess the use of podcasts among emergency medicine (EM) students, residents, and attending physicians for non-core content.

Methods

A survey was administered to medical students interested in emergency medicine, current residents of emergency medicine, and attending physicians to determine which podcasts, if any, they listen to and how often. The purpose of listening to these podcasts was also evaluated and determined it was to learn the non-core content of the specialty. The survey was administered online via multiple platforms including Google Forms (Google, Inc., Mountain View, CA), email, and Twitter (San Francisco, CA). The survey was also distributed via messaging application and word of mouth.

Results

There were 52 responses that were received on the survey. Of the respondents, 73.1% (N = 38) stated that they do listen to medical podcasts. Of these listeners, 21.2% (N = 11) stated that they listen to them once a year, 15.4% (N = 8) once a month, 11.5% (N = 6) once every two weeks, 19.2% (N = 10) weekly, 11.5% (N = 6) 1-2 days per week, 5.8% (N = 3) 3-4 days per week, 5.8% (N = 3) 5-6 days per week, and 9.6% (N = 5) daily. Based on the 52 responses received, 30.8% (N = 16) stated that they listen to them as a supplement to other sources of foundational content, 25% (N = 13) for general interest, 28.8% (N = 15) for entertainment, 7.7% (N = 4) as a primary resource to learn foundational content in their specialty, and 7.7% (N = 4) other. Based on the data metrics, 51.9% (N = 27) stated that 1-5 of the medical podcasts they listen to discuss the practice of emergency medicine, 44.2% (N = 23) stated that none do, and 3.8% (N = 2) stated that more than 10 do. The list of emergency medicine podcasts being listened to are as follows: EMplify, EM:RAP, EM Over Easy, EM Clerkship, Core EM, EM Basic, EMA, EM Cases, Internet Book of Critical Care, Pharm So Hard, EMCRIT, EM Board Bombs, PEM Rules, and REBEL CAST. When asked how important medical podcasts are for learning within the specialty, 44.2% (N = 23) reported not important, 26.9% (N = 14) reported somewhat important, and 28.8% (N = 15) reported very important. When asked how important medical podcasts are for learning outside the specialty, 40% (N = 20) reported not important, 48% (N = 24) reported somewhat important, and 12% (N = 6) stated very important. When asked if their program includes medical education podcasts as part of their curriculum, 18.4% (N = 9) responded yes, while 81.6% (N = 40) responded no. When asked if they believe medical education podcasts should be included as part of their curriculum, 52% (N = 26) stated yes, while 48% (N = 24) stated no.

Conclusion

Based on the results of the survey, roughly three-quarters of the surveyees listen to medical podcasts. Of the respondents, 38.5% (N = 20) stated that they listen to these podcasts as some form of supplement to learning content (7.7% (N = 4) primary and 30.8% (N = 16) supplementary), which explains the rising availability of the number of podcasts related to emergency medicine. Of the respondents stating that medical podcasts should be included in their curriculum, 52% (N = 26) signifies the importance of quality learning that these medical podcasts provide to their listeners.

## Introduction

Podcasts have quickly become a rising trend among students in various fields as a learning resource. Discoveries from a recent survey shed light on the significance of medical podcasts for aspiring and practicing medical professionals. The majority of respondents rely on these podcasts as a valuable resource, supplemental to their foundational knowledge, proving that they provide more than just a form of entertainment. While a minority acknowledged the importance of learning within emergency medicine (EM), a staggering 52% (N = 26) believe that medical podcasts should be integrated into the standard medical curriculum. Podcasts have emerged as an increasingly popular learning resource across various educational backgrounds. A 2018 article explored the impact of podcasts in education and highlighted their ability to facilitate learning beyond the traditional classroom setting [1]. Within the realm of medical education, a 2020 survey involving 356 emergency medicine (EM) residents revealed that a remarkable 88% prefer using podcasts to enhance their learning at least once a month [2]. Additionally, a number of EM residents state that it is the most valuable use of their time [3]. Furthermore, emergency medicine boasts the highest number of active podcasts compared to other specialties [4-6]. Given the high demand and popularity of emergency medicine podcasts, it is crucial to comprehend the logistics behind their efficacy. This study aimed to explore the frequency and purpose of listening to emergency medicine podcasts and identify the most popular ones. By understanding why and how these podcasts are being consumed, we can better harness their benefits in the medical field.

## Materials and methods

A survey was conducted on Google Forms (Google, Inc., Mountain View, CA) to obtain data among medical students interested in emergency medicine, as well as emergency medicine residents and attending physicians. The survey consisted of three sections: the first focused on demographics, the second obtained basic information about medical podcast use, and the third gathered data on information about non-medical podcast use. The survey was distributed via email, the social media platform Twitter (San Francisco, CA), messaging application, and word of mouth.

The questions were reviewed by multiple personnel to ensure no grammar mistakes were present. Additionally, this guaranteed that the questions were of high quality and were easily understandable. This also verified that all possible response options were included for each question. The survey was sent out in July 2023 and was accepting responses until September 2023. The survey was anonymous but required the surveyees to be logged in via Gmail to prevent duplicate responses. Some questions in the survey were not required to ensure the accuracy of the data.

## Results

Roughly three-quarters of current and aspiring emergency medicine physicians and residents who completed the survey listen to medical podcasts. A breakdown of each question’s responses is highlighted in separate tables below, divided based on the three sections of the survey.

The demographics section of the survey (Table 1 and Table 2) identified that a majority of the surveyees were between the ages of 20 and 30 (73.1%, N = 38). There was a greater number of male respondents when compared to female respondents (61.5%, N = 32 versus 38.5%, N = 20), with MS/OMS2 students being among the highest respondents (42.3%, N = 22). More than half of the respondents did not have other advanced degrees (67.3%, N = 35), and because none of the respondents obtained a PhD, the question “If you selected PhD in the previous question, please specify the area of interest” had no responses. The highest undergraduate major/minor was biology (22.7%, N = 15), with psychology being the second highest (7.6%, N = 5). Because there was no preference for location in the United States, a question was included that recorded which area the surveyee resided in. An image of the United States map with labeled regions was provided within the survey to ensure the accuracy of data. The area with the highest number of respondents was the midwest (42.3%, N = 22), and the second highest was the southeast (26.9%, N = 14). Community was the highest training site characterization of the respondents (46.2%, N = 24), with rural being the lowest (11.5%, N = 6). Most of the respondents did not have a subspecialty of interest, with N/A being the highest response (33.3%, N = 19). Of the respondents who did have a subspecialty of interest, the highest was emergency medicine (26.3%, N = 15).

**Table 1:** Demographics of the surveyees. MS/OMS: medical student/osteopathic medical student, PGY: postgraduate year, MS: Master of Science, MPH: Master of Public Health, MBA: Master of Business Administration

Age			Gender			Medical training year			Other advanced degrees		
20-30	73.1%	N = 38	Male	61.5%	N = 32	MS/OMS2	42.3%	N = 22	None of the above	67.3%	N = 35
30-40	15.4%	N = 8	Female	38.5%	N = 20	Attending	15.4%	N = 8	MS	23.1%	N = 12
40-50	5.8%	N = 3	-			MS/OMS1	9.6%	N = 5	MPH	5.8%	N = 3
50-60	5.8%	N = 3	-			PGY2	9.6%	N = 5	MBA	3.8%	N = 2
-			-			PGY3	7.7%	N = 4	-		
-			-			MS/OMS3	5.8%	N = 3	-		
-			-			MS/OMS4	5.8%	N = 3	-		
-			-			PGY1	3.8%	N = 2	-		

**Table 2:** Additional academic details of the surveyees.

Undergraduate degree area of interest (major/minor)			Area of residence			Training site characterization			Subspecialty of interest		
Biology	22.7%	N = 15	Midwest	42.3%	N = 22	Community	46.2%	N = 24	N/A	33.3%	N = 19
Psychology	7.6%	N = 5	Southeast	26.9%	N = 14	Academic	23.1%	N = 12	Emergency medicine	26.3%	N = 15
Chemistry	6.1%	N = 4	West	15.4%	N = 8	Urban	19.2%	N = 10	Radiology	5.3%	N = 3
Biomedical science	4.5%	N = 3	Northeast	11.5%	N = 6	Rural	11.5%	N = 6	Sports medicine	5.3%	N = 3
Microbiology	4.5%	N = 3	Southwest	3.8%	N = 2	-			Pediatrics	3.5%	N = 2
Biochemistry	4.5%	N = 3	-			-			Family medicine	3.5%	N = 2
Molecular biology	4.5%	N = 3	-			-			Toxicology	3.5%	N = 2
Biological sciences	3%	N = 2	-			-			Medical education	3.5%	N = 2
History	3%	N = 2	-			-			Pain management	3.5%	N = 2
Health sciences	3%	N = 2	-			-			Military medicine	1.8%	N = 1
Bachelor of Science in Nursing	3%	N = 2	-			-			Ophthalmology	1.8%	N = 1
Public health	3%	N = 2	-			-			Cardiothoracic surgery	1.8%	N = 1
Biomedical engineering	1.5%	N = 1	-			-			Critical care	1.8%	N = 1
Nanoscale science	1.5%	N = 1	-			-			Pediatric emergency medicine	1.8%	N = 1
Nutrition	1.5%	N = 1	-			-			Brain/spinal cord injury	1.8%	N = 1
Healthcare administration	1.5%	N = 1	-			-			Anesthesia	1.8%	N = 1

The second section of the survey gathered information on medical podcast use (Tables 3-7). It identified that 73.1% (N = 38) of the surveyees listen to medical podcasts, while 26.9% (N = 14) do not. Of the surveyees, 21.2% (N = 11) stated that they listen to medical podcasts at least once a year, with the second highest (19.2%, N = 10) stating that they listen to them weekly. When asked the reasoning for listening, 30.8% (N = 16) stated that they listen for the purpose of supplementing other sources of foundational content. Of the surveyees, 51.9% (N = 27) stated that approximately 1-5 of the medical podcasts they listen to discuss the practice of emergency medicine. The most popular emergency medicine podcast among the surveyees was EM:RAP (19.7%, N = 13), with the second most popular being EM Over Easy (15.2%, N = 10). Since this was not a required question, only 26 responses were recorded. Of the surveyees, 26.9% (N = 14) stated that medical podcasts are somewhat important for their learning within emergency medicine; however, 48% (N = 24) stated that medical podcasts are somewhat important for their learning outside of emergency medicine. Since the question asking if medical podcasts were important for their learning outside of emergency medicine was not a required question, only 50 responses were recorded. A majority of the surveyees (44.9%, N = 22) stated that the optimal length of a medical podcast is between 15 and 30 minutes, and 34.7% (N = 17) stated that 31-60 minutes is optimal. Since this was not a required question, only 49 responses were recorded. A majority of the surveyees stated that they are driving when listening to medical podcasts (56%, N = 28). Since this was not a required question, only 50 responses were recorded. Only 18.4% (N = 9) of the responders indicated that medical podcasts are included as part of their medical curriculum, with 52% (N = 26) stating that they believe medical podcasts should be included as part of their medical curriculum. A majority of the surveyees stated that they discovered these medical podcasts by word of mouth from other residents/medical students/attendings (31%, N = 31). Since this was not a required question, only 47 responses were recorded.

**Table 3:** Details of podcast usage and purpose among surveyees.

Medical podcast usage			Frequency of medical podcast listening			Purpose for medical podcast listening		
Yes	73.1%	N = 38	Once a year	21.2%	N = 11	Supplement other sources of foundational content	30.8%	N = 16
No	26.9%	N = 14	Weekly	19.2%	N = 10	Entertainment	28.8%	N = 15
-			Once a month	15.4%	N = 8	General interest	25%	N = 13
-			Once every 2 weeks	11.5%	N = 6	Primary resource to learn foundational content in my specialty	7.7%	N = 4
-			1-2 days per week	11.5%	N = 6	Other	7.7%	N = 4
-			Daily	9.6%	N = 5	-		
-			5-6 days per week	5.8%	N = 3	-		
-			3-4 days per week	5.8%	N = 3	-		

**Table 4:** Details of emergency medicine-specific podcasts among surveyees.

# of medical podcasts discussing emergency medicine			Name of emergency medicine podcasts			Medical podcast importance within emergency medicine			Medical podcast importance outside of emergency medicine		
1-5	51.9%	N = 27	EM:RAP	19.7%	N = 13	Not important	44.2%	N = 23	Somewhat important	48%	N = 24
None	44.2%	N = 23	EM Over Easy	15.2%	N = 10	Very important	28.8%	N = 15	Not important	40%	N = 20
More than 10	3.8%	N = 2	EM Cases	10.6%	N = 7	Somewhat important	26.9%	N = 14	Very important	12%	N = 6
-			EMCrit	9.1%	N = 6	-			-		
-			EM Basic	9.1%	N = 6	-			-		
-			Core EM	9.1%	N = 6	-			-		
-			EM Clerkship	7.6%	N = 5	-			-		
-			EM Board Bombs	3%	N = 2	-			-		
-			ERcast	1.5%	N = 1	-			-		
-			Undifferentiated Medical Student	1.5%	N = 1	-			-		
-			The Gifted Life	1.5%	N = 1	-			-		
-			Stimulus	1.5%	N = 1	-			-		
-			EMRA	1.5%	N = 1	-			-		

**Table 5:** Logistics of medical podcasts and activities of surveyees while listening.

Optimal length of medical podcasts			Primary activity while listening		
15-30 minutes	44.9%	N = 22	Driving	56%	N = 28
31-60 minutes	34.7%	N = 17	Chores	20%	N = 10
<15 minutes	18.4%	N = 9	Exercise	14%	N = 7
>2 hours	2%	N = 1	Other	8%	N = 4
-	-	-	Dedicated study time	2%	N = 1

**Table 6:** Inclusion of medical podcasts in the standard curriculum.

Medical podcasts as part of the standard curriculum			Potential medical podcast inclusion in standard curriculum		
No	81.6%	N = 40	Yes	52%	N = 26
Yes	18.4%	N = 9	No	48%	N = 24

**Table 7:** Details on how surveyees became aware of the podcasts they listen to.

How do you find the medical podcasts you listen to?		
Word of mouth from other residents/medical students/attendings	31%	N = 31
Internet search	25%	N = 25
Recommendation from a lecturer or faculty member	17%	N = 17
Apple Music/Android search	13%	N = 13
Other	4%	N = 4

The last section of the survey asked respondents questions regarding their use of non-medical podcasts (Table 8). Of the surveyees, 88.5% (N = 46) stated that they do listen to non-medical podcasts, while 11.5% (N = 6) stated that they do not. Of the surveyees, 23.1% (N = 12) stated that they listen to them at least once a year. Table 8 displays which non-medical podcasts surveyees listen to. While the distribution of the different podcasts being listened to was not significant, Crime Junkie was the most popular (4.3%, N = 4). Since this was not a required question, only 38 responses were recorded. Due to the volume of podcasts recorded, only podcasts with a percentage greater than 2% are included in Table 8.

**Table 8:** Details of Non-medical podcast usage among surveyees.

Non-medical podcast usage			Frequency of non-medical podcast listening			Name of non-medical podcasts		
Yes	88.5%	N = 46	Once a year	23.1%	N = 12	Crime Junkie	4.3%	N = 4
No	11.5%	N = 6	Weekly	15.4%	N = 8	JRE	3.2%	N = 3
-			Once a month	13.5%	N = 7	The Deck	2.2%	N = 2
-			Daily	13.5%	N = 7	Joe Rogan	2.2%	N = 2
-			Once every 2 weeks	11.5%	N = 6	Stuff You Should Know	2.2%	N = 2
-			1-2 days per week	9.6%	N = 5	Freakonomics	2.2%	N = 2
-			3-4 days per week	7.7%	N = 4	The American Life	2.2%	N = 2
-			5-6 days per week	5.8%	N = 3	-	-	-

## Discussion

The majority of respondents listen to medical podcasts for a variety of reasons. The data revealed that the primary purpose for listening is to supplement foundational knowledge (N = 16). This highlights the value of medical podcasts beyond strictly entertainment. Interestingly, 44.2% (N = 23) of respondents stated that medical podcasts were not important for learning within emergency medicine; however, 48% (N = 24) stated that medical podcasts were somewhat important for learning outside of emergency medicine. This data point poses the likelihood that medical students and aspiring physicians who are not interested in pursuing emergency medicine may still be listening to emergency medicine podcasts, further contributing to their success and popularity. Another question in the survey that asked surveyees to indicate their specialty of interest furthers this likelihood, as there were respondents who had indicated fields other than emergency medicine, most notably radiology (N = 3) and sports medicine (N = 3). Furthermore, a significant 52% (N = 26) believe that medical podcasts should be incorporated into standard medical curriculums. This underlines the importance of medical podcasts for medical students, residents, and attending physicians as a learning resource. However, it is important to note some limitations of this study. There may be response bias, leading to inaccurate answers that could have influenced the data. Various factors, such as disinterest or misunderstanding of questions, could have contributed to these inaccurate responses. Close-ended questions with limited response options may have also restricted the depth of information gathered. The survey did not evaluate which group of surveyees (medical students, residents, or attending physicians) listen to these podcasts the most, which is a data point that can be gathered in a future study. Additionally, the sample size was relatively small (N = 52), which can hinder its generalizability. It is important to consider these limitations when interpreting the data.

Notably, the popular medical podcast among survey participants is the emergency medicine podcast, EM:RAP. A previous study conducted from 2015 to 2016 also identified it as the most popular among emergency medicine residents [7]. This signifies the type and quality of content delivered in this podcast based on its consistent popularity for the past nine years. Furthermore, while emergency medicine has the most number of active podcasts [4-6], pediatric emergency medicine podcasts are lacking and vastly underrepresented compared to other specialties [8].

Two tools, the Academic Life in Emergency Medicine Approved Instructional Resources Series (ALiEM AIR) and Approved Instructional Resources - Professional Series (AIR-Pro), were developed in 2014 and 2015, respectively [9]. These tools were developed in response to the rising use of online blogs and podcasts as a means for educating emergency medicine residencies [9]. The ALiEM is a reliable, five-question tool used to rate online emergency medicine resources by medical educators [10]. The purpose of both the AIR and AIR-Pro series is to promote online education by providing a resource for emergency medicine residencies to evaluate which online blogs and podcasts are of high quality for their residents [11,12]. The rubric consists of five measurements based on seven-point Likert scales, which include recency, accuracy, educational utility, evidence-based, and references [13]. Posts that earn a total number of points greater than or equal to 30 out of 35 receive an AIR badge [14]. Since its creation, these tools have been used to objectively assess the quality of these rapidly increasing blogs and podcasts. A study published in 2020 utilized these tools to evaluate 61 posts specifically on infectious diseases, while another study published in 2019 reviewed 78 posts relevant to gastrointestinal emergencies [9,15]. These tools ensure that the vast number of emergency medicine podcasts that are available consist of high-quality and relevant content.

## Conclusions

The survey results reveal that medical podcast listeners find them valuable for various reasons, particularly as a supplement to other learning materials. This emphasizes the informative nature of medical podcasts beyond entertainment. Interestingly, while only a minority of survey participants consider medical podcasts highly important for learning within emergency medicine, a majority believe that they should be included in the standard medical curriculum. This highlights the significance of medical podcasts for medical students, residents, and attending physicians.

Further research is needed to delve into the specific aspects of medical podcasts that listeners find most beneficial for their learning. Understanding these gaps in medical education that podcasts can fill would be valuable. Notably, the popular medical podcast among survey participants is the emergency medicine podcast, EM:RAP. More research is necessary to decipher the content and delivery methods that make EM:RAP so valuable and popular among listeners.

## Appendices

Table 9 presents the questions in the three sections of the survey and their responses.

**Table 9:** Questions in each section and their responses to the survey. MS/OMS: medical student/osteopathic medical student, PGY: postgraduate year, MS: Master of Science, MPH: Master of Public Health, MBA: Master of Business Administration, JD: Juris Doctor, MPA: Master of Public Affairs, PhD: Doctor of Philosophy

Section 1: Demographics	Section 2: Medical podcast use	Section 3: Non-medical podcast use
Age	Do you listen to medical podcasts?	Do you listen to non-medical podcasts?
20-30	Yes	Yes
			30-40
40-50			
			No	No
50-60				
	60+			
Gender		How often do you listen to podcasts?			How often do you listen to non-medical podcasts?
Male	Once a year	Once a year		
	Once a month	Once a month
Female	Once every 2 weeks	Once every 2 weeks
	Weekly	Weekly
Other	1-2 days per week	1-2 days per week
	3-4 days per week	3-4 days per week
Prefer not to answer	5-6 days per week	5-6 days per week
	Daily	Daily
Medical training year	Why do you listen to podcasts?	If yes, please list the names of the podcasts (fill-in).
MS/OMS1	Primary resource to learn foundational content in my specialty	-
			MS/OMS2
				Supplement other sources of foundational content
					MS/OMS3
						Entertainment
							MS/OMS4
								PGY1
General interest								
			PGY2					
									Community involvement
	PGY3								
					PGY4				
				Connection with others					
							Fellow		
						Other			
			Attending						
								Other advanced degrees		How many of the podcasts you listen to discuss the practice of emergency medicine?	-
MS	None							-	
					MPH				
									1-5
							JD		
MPA									
			6-10						
				MBA					
	PhD								
More than 10									
					None of the above			
				If you selected PhD in the previous question, please specify the area of interest (fill-in).		List the name of the emergency medicine podcasts you listen to (fill-in).		
	Undergraduate degree area of interest (major/minor) (fill-in)			How important are medical podcasts for your learning within emergency medicine?		-		
	-		Not important	-				
			Somewhat important					
			Very important					
In which area of the United States do you live (image included)?	How important are medical podcasts for your learning outside of emergency medicine?		-					
Northeast	Not important		-					
				Southeast				
					Somewhat important			
Southwest								
				West				
	Very important							
Midwest								
				How would you characterize your training site?	What is the optimal length of a medical podcast?	-		
				Community	<15 minutes, 15-30 minutes, 31-60 minutes, 1-2 hours, >2 hours	-		
15-30 minutes								
	Academic							
							31-60 minutes	
					Rural		
1-2 hours							
				Urban			
	>2 hours					
			Subspecialty of interest (fill-in)				What is the primary activity you do while listening to medical podcasts?	-
-			Exercise		-	
			Chores			
	Driving					
	Dedicated study time					
	Other					
-	Does your program include medical education podcasts as part of your standard curriculum?		-			
-	Yes		-			
	No					
-	Do you think medical podcasts should be included as a part of your standard curriculum?		-			
-	Yes		-			
	No					
-	How do you find the medicine podcasts you listen to? Select all that apply.		-
-	Word of mouth from other residents/medical students/attendings		-
	Apple Music/Android search		
	Internet search		
	Recommendation from a lecturer or faculty member		
	Other		
